# The effects of economic development and built environment on diabetes in CHINA

**DOI:** 10.1186/s12963-017-0152-2

**Published:** 2017-09-29

**Authors:** Tao Zhang

**Affiliations:** School of Public Administration, Macao Polytechnic Institute, Macao, China

**Keywords:** Built environment, Diabetes, Estimation, Obesity, Regression, China

## Abstract

**Background:**

With rapid economy growth, the prevalence of obesity, and related chronic diseases, has increased greatly. Although this has been widely recognized, little attention has been paid to the influence of built environment and economic growth, particularly for developing countries. The main purpose of this study is to investigate the potential relationship between the prevalence of diabetes and the built environment while considering the effects of socioeconomic change in China.

**Methods:**

Three nationally representative samples are constructed and employed mainly based on various sources of data, such as the China National Nutrition and Health Survey, World Health Organization, and China Health and Retirement Longitudinal Survey in 2013. The explanatory variables representing the built environment and influential factors include the health outcomes, economic indicators, local health facilities, regional dummies, and demographic features. OLS, robust regressions, and a set of binary choice models are used to estimate the possible relationship.

**Results:**

It is suggested that the prevalence of diabetes is associated with both the broader built environment and individual economic factors in China. China’s sharp economic growth in the recent decades has greatly increased the prevalence of obesity and diabetes, when also considering other influential factors.

**Conclusions:**

Although the results can not specify causal mechanism, some useful results can be clearly discovered and subsequently a few important policy implications can be provided for the sustainable and healthy development of China’s urban planning or built environment.

## Background

Obesity, triggered by excessive body fat, has reached pandemic levels and thus influences population health seriously. As a severe medical and social problem, obesity poses a harsh threat to public health for its potential association with a bundle of chronic diseases such as diabetes and hypertension. In recent decades, the risk of obesity and related chronic diseases have been intensively studied [1–4]; however, obesity and related chronic diseases in developing countries have been relatively neglected [5], though few developing countries are unaffected by these conditions. China, as a developing country, is experiencing a sharp economic growth and subsequently, a substantial increase in weight level, and is receiving an increasing amount of attention [6–8]. While diabetes is a main source of risk for cardiovascular disease, which has become the leading cause of death in China, the prevalence of diabetes is greatly increasing as an obesity epidemic occurs in tandem with the rapid economic growth of the past decades [9–12]. This study explores the association of diabetes and its relationship with both the built obesogenic environment and individual factors in China. While most of the existing literature on this topic only concerns the role of individual-level environmental factors, the influence of the broader built environment has attracted more attention. The term built environment here refers to the man-made surroundings that influence the prevalence of obesity, including physical design, residential, economic, and other activities, and local health facilities. It is suggested that diet-related health outcomes, such as obesity and diabetes, cannot fully be examined on the individual level [13, 14]. The related living activities and the consequent health outcomes requires a full understanding of both the environment and individual-level activity. Swinburn et al. [15] proposed that, as individuals interact with the environment on a number of levels, both the microenvironment and broader macroenvironment can determine the individual’s behavior and health outcomes. Therefore, successful policy interventions depend on the extent of both the macroenvironment and microenvironment associated with obesity and diabetes. However, most of the available literature focuses only on individual-level analysis. There are not many observed studies examining the macroenvironment or built environment and the association with China’s obesity and diabetes epidemic. This paper highlights the importance of the macroenvironment, while considering individual-level factors. A nationally representative dataset was constructed and employed on the community level. County-level data of health outcomes were collected from China National Nutrition and Health Survey 2002 (CNNHS 2002). The data for the built environment were collected from China County Statistical Yearbook 2002 and China Statistical Yearbook for Regional Economy 2002. Then, the data were combined from these three surveys to form a new dataset specifically designed for community-level analysis. In addition to the above data, data were also employed from the Study on Global Ageing and Adult Health conducted by WHO for comparison. The prevalence of diabetes in Russia and India is used as a comparison to China in order to provide additional information for inference. The individual-level data from China Health and Retirement Longitudinal Survey in 2013 are utilized for microenvironment investigation, which subsequently provides up to date information and supports multilevel binary analysis. Because all the used data from different surveys are randomly collected, they should be representative for China. Thus, we can compare them for different years in the analyses.

## Methods and data

### Data for community-level investigation

CNNHS collected a nationally representative sample from all 31 provinces of China, using stratified random sampling (*N* = 272,023). CNHHS randomly chose 132 counties from these 31 provinces^1^ to gather the 272,023 observations (*N* = 272,023). The effects of the built environment on health outcomes can have a variety of forms, from physical activity and food option to socioeconomic status and regional living patterns. It should be noted that the definitions for overweight, underweight, and obesity are varied across different countries. As it is widely recognized that people of Asian-Pacific populations have a higher risk for obesity-related diseases than Caucasians at the same level of body mass index (BMI) [16–18], WHO and Asian-Pacific Consensus Statement recommend a lower BMI cutoff point (at 25 instead of 30) for the definition of obesity in China. Accordingly, the standards were proposed in conformity to the fact that in China a BMI of 28 (equal to or higher than 28) was discovered to have the best sensitivity and specificity for identifying risk factors for obesity-related chronic diseases [16]. Consequently, the obesity rate in this study is defined as the percentage of persons with BMI higher than 27.

**Fig. 1:**
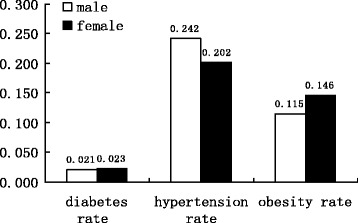
The prevalence of diabetes, obesity, and hypertension stratified by gender

**Fig. 2:**
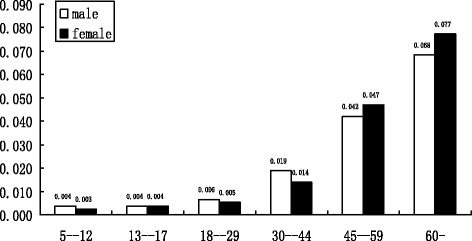
The prevalence of diabetes stratified by gender and age groups

The diabetes rate is the percentage of persons (age > 5) with diabetes. The hypertension rate is the percentage of persons (age > 15) with high blood pressure. The main purpose of this study is specifically designed to estimate the prevalence of diabetes and the influence of the built environment including economic indicators, health facilities, regional disparities, and demographic features. The local averaged GDP is computed by dividing regional GDP by the number of people in the local population, indicating the GDP per capita. Local average wage is measured in the same way as GDP. The household size is measured by the number of family members residing in the home. The health center density is the number of local health centers per 10,000 persons and hospital density is the number of local hospitals per 10,000 persons. In addition, regional dummies are also considered in the estimation. The regional dummies are used to capture the potential enormous regional disparities over the whole of China. One dummy variable is employed to represent the north-south gap as traditional China could be divided into two sections (north-south) by the Yangzi River. The other two dummies are used to indicate the east-middle-west disparity as these three sections of China normally have different developing levels. Figure 1 depicts the prevalence of obesity and related chronic diseases including diabetes and hypertension. The data in Fig. 1 are calculated and estimated from the county-level regional data collected from CNNHS 2002 and show a large number of people in China affected by obesity and related chronic diseases. The obesity rates of male residents and female residents in China in 2002 were 11.5% and 14.6% respectively, indicating that women were more likely to be obese than men. The prevalence of hypertension for male residents was relatively higher than that of female residents (24.2% vs. 20.2%, respectively). The prevalence of diabetes in China in 2002 was about 2.2% overall, while the diabetes rate of male residents was lower than female residents (2.1% vs. 2.3%, respectively). This result shows that the prevalence of obesity had increased greatly, by 80.6% between 1992 and 2002 [19]. Figure 2 describes the prevalence of diabetes among men and women by age groups. It is indicated that, among the age group from 5 to 12 years, the diabetes rate was only 0.36% for men and 0.26% for women. For those aged 13–17 years, the prevalence of diabetes among men was 0.39% and 0.36% among women. The prevalence of diabetes for the group aged 18–29 years was 0.64% for men and 0.54% for women, respectively. The prevalence of diabetes for the group aged 30 to 44 years more than doubled compared to the previous age group, as 1.87% of men and 1.39% of women had diabetes. Among the age group from 45 to 59 years, this trend continued as the diabetes rate for men more than doubled to 4.22% and for women more than tripled to 4.7%. For the age group over the age of 60 years, the prevalence of diabetes for men increased further to 6.82% and 7.72% for women. A potential threshold was available between the age group 18–29 and 30–44, which triggered an explosive increase in diabetes prevalence. Additionally, a sudden change existed between the age group 30–44 and 45–59 where women were shown to have a higher diabetes rate than men, despite having a lower diabetes rate than men before this age change point. Subsequently, it is apparent that younger males are more likely to be diabetic than younger females while elderly females are more likely to be diabetic than elderly males in China.

The summary statistics of variables contained in the regression for the estimation of the relationship between diabetes and influential built environment are listed in Tables 1 and 2. Table 1 lists the summary statistics of all the health outcome variables stratified by gender. It shows that the average ages for the men and women included in the survey are close to each other, indicating high comparability of the data for men and women. The results in Table 1 reinforce the results in Fig. 1, further indicating that obesity and diabetes are more prevalent in women than men while hypertension is more likely to be prevalent in men rather than women in China. In the regression, with diabetes as a dependent variable, the obesity rate and hypertension rate are also contained as possible explanatory variables to probe the potential relationship between them. Additionally, it is proposed by some previous studies that obesity and diabetes probably share a simultaneous relationship and they suggest that obesity may cause diabetes [20–23]. This study tries to examine the linkage between obesity (and hypertension) and diabetes, though the causality mechanism can not be easily interpreted in this study since it employs cross-sectional data. Table 2 depicts the summary statistics of built environment variables which are employed in the estimation to investigate the possible association between diabetes and a macro-level built environment. The average household sizes for 132 county-level regions ranged from 2.6 persons to 4.47 persons with a mean at 3.52 persons, showing a relatively small household size in China as a whole. Health center density ranged from 0.39 to 7.08 with a mean at 1.95, indicating that the number of local health centers per 10,000 persons changed greatly across 132 regions. Furthermore, this phenomenon represents unbalanced levels of development of health facilities in different regions in China. Hospital density also varied greatly, ranging from 0.09 to 3.17. This means that in a region with a well-built health environment, about 3155 people could access one hospital, but in a region with a badly built health environment, 10,000 people could access only one hospital between them. Such enormous disparity in different regions would certainly result in various health outcomes. The GDP per capita ranged from 1312 to 47,053 RMB with the mean at 10,825.74, indicating that the economy in different regions over the whole of China was developed extremely unevenly. The average wage per capita for 132 regions ranged from 2624 to 28,589 RMB with the mean at 10,752.64, again representing uneven income levels across different regions.

**Table 1:** Summary statistics of county-level health variables (132 regions)

Variables	Mean	Std. Dev.	Min	Max
Health outcome variables for men				
Diabetes rate (%)	2.11	2.34	0.00	14.36
Obesity rate (%)	11.45	7.92	1.10	36.73
Hypertension rate (%)	24.18	9.46	7.99	46.20
Average age	44.86	3.42	37.76	55.24
Health outcome variables for women				
Diabetes rate (%)	2.31	2.41	0.00	14.73
Obesity rate (%)	14.62	9.02	0.93	38.30
Hypertension rate (%)	20.22	7.82	3.67	42.12
Average age	42.54	2.84	36.21	50.86

Note: The data here are calculated and estimated from the county-level regional data in CNNHS2002

**Table 2:** Summary statistics of county-level variables for built environment (132 regions)

Variable	Mean	Std. Dev.	Min	Max
Household size (number of household members)	3.52	0.40	2.60	4.47
Health center density	1.95	1.41	0.39	7.08
Hospital density	0.58	0.35	0.09	3.17
Average GDP (RMB)	10,825.74	10,395.33	1312.00	47,053.00
Average wage (RMB)	10,752.64	4012.60	2624.00	28,589.00

Note: The data here are calculated and estimated from the county-level regional data in CNNHS2002

This study employs multi-variable regressions to investigate the potential relationship between the regional diabetes rate and influential factors of a broader built environment. The method of ordinary least squares (OLS) is used since cross-sectional data are employed. However, when a skewed distribution is present, the OLS assumption will not be satisfied by the non-normal error distribution. As is well known, the above phenomenon will seriously impair the efficiency of OLS estimates and most likely result in heavily biased estimation. To solve this problem, this study utilizes three non-normality tests to detect the potential non-normal distribution. Then, robust estimation is used to accommodate the skewed distribution if it is available. Yohai [24] proposed a method attempting to combine the resistance of S-estimation first introduced by Rousseeuw & Yohai [25] and the efficiency of M-estimation proposed by Huber [26]. Therefore, this method, also known as robust estimation, obtains the resistance to skewed distribution, whilst retaining efficiency. Consequently, this study employs the robust estimation to account for the potential non-normality and outliers.

### Data for individual-level investigation

**Fig. 3:**
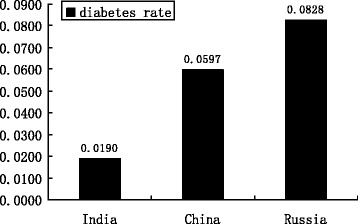
The prevalence of diabetes in India, China, and Russia (from 2007 to 2010)

WHO and SAGE conducted the Study on Global Ageing and Adult Health from 2007 to 2010 in several countries. For the purpose of comparison, this paper chooses Russia and India to be compared with China’s diabetes prevalence. Figure 3 depicts the prevalence of diabetes in India, China, and Russia. Figure 3 shows that the diabetes rate was only 1.9% for India as a whole in 2007, compared to the prevalence of diabetes in China at 6% and 8.28% in Russia. This enormous disparity may come from the geographical difference, the genetic diversity, and the distinction in social and economic development.

**Fig. 4:**
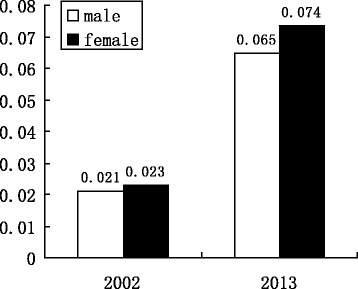
The prevalence of diabetes stratified by gender in 2002 and 2013

**Fig. 5:**
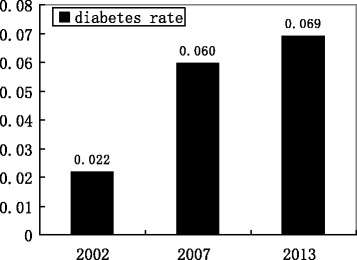
The prevalence of diabetes in 2002, 2007, and 2013

The China Health and Retirement Longitudinal Survey in 2013 provided up-to-date information for individual-level analysis for diabetes prevalence and its influential factors. Figure 4 describes China’s prevalence of diabetes stratified by gender in 2002 and 2013. It shows that the diabetes rate for males increased from 2.1% in 2002 to 6.46% in 2013, whereas the prevalence of diabetes for females increased from 2.3% to 7.36% during the same period. The difference of diabetes rate between males and females was significantly larger in these five years. Figure 5 provides the prevalence of diabetes in China in 2002, 2007, and 2013. Between 2002 and 2013 the prevalence of diabetes shows an upward trend, indicating that more people in the population are developing diabetes. Due to the severe status of this pandemic disease, this should be investigated further. This paper will use individual-level data in 2013 to explore the association between microenvironment factors and the prevalence of diabetes.

The China Health and Retirement Longitudinal Survey in 2013 (CHARLS) also collected a nationally representative sample with *N* = 18,617. Again, it used the stratified random sampling method in the survey. Thus, CHARLS covered 28 of the 31 provinces in China and randomly chose 126 cities from those provinces. In these cities, about 450 communities were randomly chosen. Finally, 18,617 randomly selected observations formed the representative sample. To investigate the relationship between diabetes and individual-level factors, several explanatory variables were selected in the binary choice regressions, including obesity status (having or not having obesity as reported by individuals), hypertension status (having or not having hypertension as reported by individuals), marriage status, gender, individual income per month (measured in Yuan RMB), and house building area (measured by square meters). Table 3 lists the frequency and percent of all binary choice dummies. Table 4 describes the summary statistics of continuous variables including income and house building area. From 13,169 valid observations, 1930 people were obese, occupying 14.66% of total valid observations. It showed that the obesity rate of all China increased from nearly 13% to 14.66% in 11 years. The number of valid observations for hypertension was only 2747 and 598 of these reported that they had hypertension, indicating a hypertension rate at 21.77% which was similar to the hypertension rate in 2002. About 2433 people of 18,585 valid observations were single, occupying 13.09% of all valid observations. As for gender, 47.77% of all valid observations were male, and the remaining were female. Individual income per month, as a continuous variable, ranged from 8.3 to 17,000 with a mean at 1990.9, indicating an enormous disparity between rich and poor people in China in 2013. The variable of house building area also changed substantially, ranging from six square meters to 800 square meters with a mean at 120.456. In Tables 3 and 4, the dummy variable of diabetes is used as the dependent variable and all the others are independent.

**Table 3:** Frequency of individual binary variables for influential factors (18,617 observations in 2013)

Variable	Frequency of 1/total valid observations	Percent
Diabetes (0 no/1 yes)	190/2745	6.92%
Obesity (0 no/1 yes)	1930/13169	14.66%
Hypertension (0 no/1 yes)	598/2747	21.77%
Marriage (0 married/1 single)	2433/18585	13.09%
Gender (0 female/1 male)	8798/18418	47.77%

Note: The data here are calculated and estimated from the individual data in China Health and Retirement Longitudinal Survey 2013

**Table 4:** Summary statistics of influential variables for individuals (18,617 observations in 2013)

Variable	Mean	Std. Dev.	Min	Max
Individual income per month (Yuan RMB)	1990.9	1749.248	8.333	17,000
House building area (squared meters)	120.456	79.180	6	800

Note: The data here are calculated and estimated from the individual data in China Health and Retirement Longitudinal Survey 2013

## Results

### Results of county-level regressions using data in 2002

The robust estimation is used to account for the potential non-normality and outliers. Because the dataset is multi-level, the regional effects may exist. Therefore, the inter-county correlations are also considered for the main explanatory variables being at the county level. The standard errors are adjusted for 132 clusters by using county-level variables (such as local GDP). The result shows that there is no significant systematic difference between the clustered standard errors and the standard errors estimated by the common methods. Since the results of OLS and robust estimation have been listed in Table 5, the clustered results need not to be provided.

**Table 5:** Estimated results for regressions using diabetes as dependent variable

Variable	OLS	Robust Estimation
Obesity rate	0.1329***	0.0981***
Hypertension rate	0.0279	0.0221*
Ln(GDP)	0.0036*	0.0023*
Ln(wage)	0.0066	0.0051*
Health center density	0.0037***	0.0023***
Hospital density	−0.0063*	−0.0055**
Household size	−0.0064*	0.0004
Gender	−0.001	−0.0005
Age	0.0022***	0.0013***
East	0.0064*	0.0031*
Middle	0.0008	0.0007
North	0.0024	0.0014
Constant	−0.0152	−0.0709***
F (Wald) test for all variables	24.07***	37.60***
R square	0.5124	
Adjusted R square	0.4911	
Hausman Test for two regressions	χ^2^-statistic—46.22***	
Skewness/Kurtosis test for Normality	χ^2^-statistic—18.81***	
Shapiro-Wilk W test for Normality	z-statistic—8.615***	
Shapiro-Francia W test for Normality	z-statistic—7.829***	

Note: The null hypothesis for F or Wald test is that the concerned coefficients are jointly equal to zero. The null hypothesis for normality test is the normal distribution of the model

*, **, and *** give the coefficients’ significance indicated by estimated standard errors or bootstrap standard errors at 10%, 5% and 1% level individually

In the diabetes regressions, the independent variables, accounting for built environment, include county-level socioeconomic factors (e.g., natural logarithms of the local GDP per capita and the local averaged wage), health facility factors (e.g., hospital density and health center density), demographic features (e.g., household size, regional average age and county-level gender divisions) and other health outcomes probably influencing diabetes (e.g., obesity and hypertension). According to the estimated results in Table 5, it is clear that the F-statistics of Wald test for both OLS and robust estimation are statistically significant indicating that all the independent variables should be contained in the regression equations. According to three non-normality tests depicted in Table 5, it is obvious that the normality hypotheses are all significantly rejected. Consequently, considering the non-normality of data distribution, the estimators of robust regression are asymptotically sound. Additionally, the Hausman test is executed to detect the systematic differences between OLS estimators and robust estimators. The test provides the χ^2^ statistic at 46.22 which significantly rejects the original hypothesis and again indicates the estimators of robust estimation should be preferred. According to the estimated results from both OLS and robust estimation in Table 5, local GDP per capita is shown to be significantly (statistically at the 10% level) related to the prevalence of diabetes. Because of the positive coefficients estimated, it is clear that an increase in GDP is associated with a higher diabetes rate in China. Although the OLS estimated coefficients on the average wage are statistically insignificant, it is statistically significant at the 10% level for robust estimation indicating that the increasing wage may have a positive relationship with the prevalence of diabetes. While there are fewer observed studies on diabetes and socioeconomic indicators in China, the results here suggest that the local economy growth is positively associated with regional diabetes rate. The estimate of averaged household size from OLS is negative and statistically significant at the 10% level, though the robust estimation yields a positive and insignificant estimate. This significant estimate from OLS indicates that a diminishing household size may have a positive effect on reducing diabetes rate.

The estimates of the gender dummy variable are insignificant for both regressions, indicating that there is no clear relationship between diabetes and gender. This is inconsistent with the significant relationship between other chronic health outcomes (e.g., obesity and hypertension) and gender. Furthermore, it shows that the gender has no intensive impact on the prevalence of diabetes. According to the estimated results of both regressions, age has an intensively significant and positive relationship with diabetes rate. This result is certainly consistent with most previous studies where a similar status was discovered. This requires that the local health policy planners should pay more attention to elderly people. The regional obesity rate has a statistically significant and positive association with diabetes rate for both OLS and robust regressions. This indicates that an increasing severity of obesity prevalence represents a higher diabetes rate on the county level in China. This performs as expected, since it is widely recognized that a people with relatively heavier weight are more likely to develop type 2 diabetes. The clear relationship between obesity and diabetes means that local governments should consider the obesity epidemic when attempting to deal with diabetes prevalence. The estimated coefficient of regional hypertension rate from robust estimation is positive and statistically significant at the 10% level, though the OLS yields an insignificant estimate. As the result of robust estimation is preferred due to the non-normality tests, this significant coefficient suggests that the hypertension may have a positive relationship with diabetes.

Hospital density has a negative and significant relationship with local diabetes rate for both regressions. This indicates that better access to the hospital can potentially reduce the local prevalence of diabetes. However, for the cross-sectional nature of the data employed here, the possibility of self-selection problem may exist as healthier people can choose to live in the communities with better access to hospitals. If this is the case, then a local policy to increase the density of hospitals would not substantially reduce the diabetes rate. Health center density, having an opposite pattern to hospital density, shows a significantly positive relationship with the diabetes rate. As this result can not identify the causality mechanism, we can not simply interpret this relationship in the way that an intensive health center density is a cause to promote the prevalence of diabetes. Again it can be cautiously interpreted as the possibility of the self-selection problem from local residents. Another potential explanation is that the local government would provide more health facilities in the areas with a higher diabetes rate in the population.

As for the regional dummy variables, only the dummy representing the location of eastern China yields the significant estimate at the 10% statistical level for both regressions. The significant and positive coefficient indicates that the local residents living in eastern China are more likely to develop diabetes. This status is rarely discussed in the previous literature. The possible interpretation is that as eastern China is more developed (in economy, transportation, and social culture) than the other sections of the country, the working pattern and lifestyle of people in this area have changed greatly. The dummy variable representing the middle of China is insignificant, showing an unclear relationship with the diabetes rate. As a result, it can be easily discovered that though the location of western China is dropped in the regression, it should have a negative relationship with the prevalence of diabetes, further indicating that the residents in the west of China have a relatively lower diabetes rate. The dummy variable representing nothern China is insignificant, indicating that the disparity of diabetes prevalence for the north and south is not clear.

### Results of individual-level regressions using data in 2013

Since the dependent variable used in this subsection is binary choice, this study employs binary choice regressions to explore the association between the diabetes dummy variable and influential factors at individual level. Except for binary choice regressions, OLS again is applied for the cross-sectional characteristics of data. The widely used binary choice regressions, such as Logit and Probit model, are applied here. However, when a skewed logit distribution is present, the common Logit model will yield inconsistent results. Therefore, we also used a skewed Logit model to address such potential problem in the data. As this study pays more attention to the community influence in the quantitative analysis, and though we utilize individual-level data in this section, the community effects still should be considered. Thus, the mixed effect Logit model is employed when we treat the community identity as the only level of nested cluster of random effects.

The estimated results of all regressions are listed in Table 6. Although the results of OLS shows to be more efficient than the other regressions, for the binary nature of employed data, we still analyze the results of Logit model rather than OLS. In fact, the results of all regressions are similar, because we believe the sign of estimated coefficients while ignoring the magnitude of them. The coefficients of obesity estimated by OLS, Logit, and mixed effect Logit are significantly positive, indicating that the people who are obese are more likely to develop diabetes. This result reinforces the significant association between obesity and diabetes which was also discovered from the data in 2002. Marriage status has a positive relationship with diabetes, whereas only OLS has significant coefficient. As 1 represents single status and 0 represents being married, the positive coefficient shows that the marriage will reduce the potential possibility to develop diabetes. This probably comes from the mutual aid and assistance in the marriage. The gender dummy variable has negative coefficients for all five regressions, whereas only OLS gives a significant estimate. This result differs from the analyzed result using data in 2002. It can be simply explained by the raw data of the prevalence of diabetes in these two years. According to Fig. 3, the disparity of the prevalence of diabetes between men and women in 2002 was negligible (only less than 0.2%), while the difference in 2013 was nearly 1%. This significant change made the effect of gender more obvious in the regressions. Hypertension is shown to have the positive effect on diabetes, which indicates that people with hypertension are more likely to develop diabetes. This result is consistent with the result from 2002 data. The individual income per month has positive, but insignificant, coefficients from all five regressions. This shows an unclear or weak positive association between income and diabetes. The house-build-area variable is shown to have a significant and negative association with diabetes. Since its coefficients from five regressions are all highly significant, such a negative relationship represents that people living in smaller house are more likely to develop diabetes. This may come from the living environment and facilities. Therefore, the government should provide better living conditions and facilities to local residents in order to reduce the prevalence of diabetes.

**Table 6:** Estimated results for binary choice model using diabetes as dependent variable

Variable	OLS	Logit	Probit	Skewed Logit	Mixed effect Logit
Obesity dummy	0.065*	0.719*	0.307	0.697	0.719*
Marriage status	0.241*	1.660	0.978	1.434	1.660
Hypertension dummy	0.056*	0.764*	0.361	0.693	0.764*
Gender dummy	−0.038*	−0.670	−0.374	−0.607	−0.670
Ln(house building area)	−0.059**	−1.300**	−0.571**	−1.247**	−1.300**
Ln(income per month)	0.013	0.329	0.187	0.301	0.329
Constant	0.239	0.570	−0.336	−12.827	0.570
F /LR(Wald) test for all variables	3.06***	14.26**	13.5**		11.74**
R square/Pseudo R square	0.67	0.14	0.14		
Adjusted R square	0.51				
Log likelihood		−42.85	−43.23	−42.62	−42.85

Note: The null hypothesis for F or LR test is that the concerned coefficients are jointly equal to zero

*, **, and *** give the coefficients’ significance indicated by estimated standard errors at 10%, 5% and 1% level individually

## Discussion

Over the past decades of high economic growth, though China’s population becomes richer and consumes more food, the public health system of China is facing a dilemma. Obesity and related chronic diseases such as diabetes are more prevalent and therefore should attract more attention from society, researchers, and the government. Due to the importance of the built environment influencing the population obesity and diabetes, it would be more reasonable to identify the built environment measures and other independent variables impacting those dependent public health variables. This study investigates the effects of the broader built environment and microscope individual factors for diabetes and subsequently indentifies the correlation between the built environment and the public health. A variety of regressions and estimations are used and compared respectively to account for the nature of data employed. A potential limitation may come from the characteristics of cross-sectional data. Many of the estimated coefficients from the variables included in the regressions can only be interpreted as co-varying with dependent variables, rather than causing them. Causality with respect to independent variables is difficult to be identified when using cross-sectional data. Moreover, in addition to the potential causal mechanism, there are two possible explanations that can be used to account for the above associations. One explanation, proposed by most previous studies, is the self-selection problem. Local residents may select the community to live for the better built environment. If this is the case, then independent variables can not be interpreted as the cause of changing in dependent variables. A second possible explanation involves the local governmental response to the public health problem in their jurisdictions. Local governments may multiply the number of health facilities or expand the magnitude of local health centers in response to solve the public health problems. The advantage of this study is that it employs both county-level data and individual-level data. As has been mentioned previously, the ineffectiveness of some government polices in absence of a supportive environment requires a broader approach to solve public health problems. This problem is especially obvious in the present China since thispublic health situation can mainly be ascribed to the high economic growth and subsequently rapid environmental change.

In addition to the limitations and advantages described above, this study includes a set of built environmental variables in a wider scope. Moreover, the potential relationship between individual factors and diabetes is also examined, which has been rarely explored on a national level in China. Furthermore, several estimation methods are used to enhance the asymptotic property of estimated results. In addition, although the results cannot specify the causal mechanism, some useful results can be clearly discovered and policy implications can be provided for local government intervention. It is clearly suggested that the environmental factors, including socioeconomic characteristics, demographic features, and public health facilities, are important explanatory variables of diabetes in China. The significant positive association between socioeconomic indicators and diabetes shows that the rapid economic growth in China will raise the risk of diabetes. This implies that the local health authorities should enhance community-level interventions to reduce the risk of diabetes and related chronic diseases, while special guidance should be given to the local residents facing high economic growth and environmental change. Since hospital density is negatively associated with diabetes, local authorities could make their efforts to expand the magnitude of regional hospitals. Better access should be achieved through effective interventions from the local authorities. However, the effectiveness of such community-level interventions has to be cautiously considered for the possible self-selection problem. The analysis also examines some demographic factors such as gender, age, and household size. An interesting result shows that generally a smaller household size may be related to a lower risk of diabetes, though it is only weakly significant. The binary choice regressions give some consistent results. Furthermore, these individual-level analyses show that government planners need to pay more attention to males as they are more likely to develop diabetes than females. The importance of the relationship between obesity and diabetes requires that local governments and planners incorporate obesity epidemic issues in public policies attempting to reduce diabetes rate and related chronic diseases. Based on these results, a local policy could then be implemented to reduce the obesity rate and substantially reduce the prevalence of diabetes.

## Conclusions

The prevalence of obesity, and related chronic diseases, has increased greatly in the recent decades in China. However, the studies on the influence of built environment and economic growth on obesity and related chronic diseases were rarely observed for developing countries. This study mainly investigates the potential relationship between the prevalence of diabetes and the changing environment in China. Based on three nationally representative samples, OLS, robust regressions, and a set of binary choice models are employed to estimate the possible association. The results show that the prevalence of diabetes is associated with both the broader built environment and individual economic factors in China. Based on these results, some useful policies of intervention can be suggested to reduce the obesity rate and substantially reduce the prevalence of diabetes.

## Abbreviations

BMI: Body Mass Index
CHARLS: China Health and Retirement Longitudinal Survey
CNNHS: China National Nutrition and Health Survey
OLS: Ordinary Least Squares
WHO: World Health Organization

## Footnotes

1 China Health and Nutrition Survey (CHNS 1989–2011) is an international collaborative project conducted by the Carolina Population Center at the University of North Carolina and the National Institute for Nutrition and Health at the Chinese Center for Disease Control and Prevention (CCDC). It collected the individual information using a multistage, random cluster process to draw a sample of over 30,000 individuals in 15 provinces in China. Therefore, CNNHS and CHNS are two different databases. CNNHS covered all 31 provinces of China and subsequently was more representative for the whole of China.
